# Gene Editing Regulation and Innovation Economics

**DOI:** 10.3389/fbioe.2020.00303

**Published:** 2020-04-15

**Authors:** Agustina I. Whelan, Patricia Gutti, Martin A. Lema

**Affiliations:** ^1^Maestría en Política y Gestión de la Ciencia y la Tecnología, Universidad de Buenos Aires, Buenos Aires, Argentina; ^2^Dirección de Biotecnología, Secretaría de Alimentos y Bioeconomía, Buenos Aires, Argentina; ^3^Departamento de Ciencia y Tecnología & Maestría en Ciencia, Tecnología y Sociedad, Universidad Nacional de Quilmes, Bernal, Argentina

**Keywords:** gene editing, innovation economy, biotechnology regulation, bioeconomy, genome editing, CRISPR-CAS, new breeding techniques, biotechnology indicators

## Abstract

Argentina was the first country that enacted regulatory criteria to assess if organisms resulting from new breeding techniques (NBTs) are to be regarded as genetically modified organisms (GMOs) or not. The country has now accumulated 4 year of experience applying such criteria, reaching a considerable number of cases, composed mostly of gene-edited plants, animals, and microorganisms of agricultural use. This article explores the effects on economic innovation of such regulatory experience. This is done by comparing the cases of products derived from gene editing and other NBTs that have been presented to the regulatory system, against the cases of GMOs that have been deregulated in the country. Albeit preliminary, this analysis suggests that products from gene editing will have different profiles and market release rates compared with the first wave of products from the so called “modern biotechnology.” Gene editing products seems to follow a much faster development rate from bench to market. Such development is driven by a more diverse group of developers, and led mostly by small and medium enterprises (SMEs) and public research institutions. In addition, product profiles are also more diversified in terms of traits and organisms. The inferences of these findings for the agricultural and biotechnology sectors, particularly in developing countries, are discussed.

## Introduction

The Argentine regulatory system for modern biotechnology applied to agriculture is recognized worldwide for being among the most experienced ones (Vicien and Trigo, 2017). Being one of the leaders in this field, in 2015 the country enacted a pioneer regulation for products of the so-called “new breeding techniques” (NBTs), including gene (or genome) editing. As described in Whelan and Lema (2015), products derived from NBTs are submitted to a case-by-case analysis in order to establish if they are genetically modified organisms (GMOs) or not. Such criteria also include cooperative links between the regulatory frameworks for GMOs and for conventional products, in order to avoid any safety or legal gap.

Technical details pertaining to scientific and legal regulatory criteria applied in this regulation can be found elsewhere, both in our recent publications (Lema, 2019; Whelan and Lema, 2019) and the updated regulatory texts (Infoleg, 2019a,b). There is also literature available that contextualizes this regulatory approach at the international level (Duensing et al., 2018; Eriksson et al., 2019; Metje-Sprink et al., 2020).

The study presented here further explores the implications for economic innovation of such regulatory activity in Argentina, by analyzing the profile of traits and organisms modified by NBTs that have been presented to the regulatory system. Although there is plenty of literature available about the impacts of GMO cultivation in Argentina and elsewhere (Brookes and Barfoot, 2018a, b, and references therein), it is not the same case for products derived from gene editing. Therefore, as we and others have discussed previously (Whelan and Lema, 2017; Maaß et al., 2019), from a policymaking perspective there is a need for studies pertaining to the potential socioeconomic impacts of gene editing applied to agriculture, including any modulatory effect that regulatory approaches can have on such impacts.

## The Role of Regulation in Innovation Processes

“Regulation” understood as the laws, norms and rules that order an economic, social or institutional process is essential to guide the technological development of countries, among other factors that also affect innovation processes. In a productive sector based on biological processes, such as the agroindustry sector, regulation is a tool that should be used to preserve the “welfare,” in the broadest sense, of society as it adopts innovations. In other words, the enactment and application of regulations is part of policymaking, where the aim is to establish frameworks for safe and adequate development within the innovation system.

As a source of codified knowledge, regulations have a direct impact on technology diffusion because they affect the generation of new technologies, as well as decisions on their adoption by potential users (OECD, 1996; Geroski, 2000). In regards to technology development, regulations have similar properties to those of a “public good”; in that the main characteristics should be “openness” and “credibility.” “Openness” refers to the situation of a regulation being accessible and applicable to all competitors, which is particularly important for small innovative companies because it grants certainty for market access. In addition, “credibility,” refers to the State being able to create confidence that a norm is of general use (Temple, 2005).

Regarding the effect on potential adopters (i.e., developers and users) of a technology, the establishment of a regulation reduces uncertainty about technological characteristics by increasing the availability of information. Therefore, it facilitates their decision process (Kat and Oomen, 2007) and the diffusion of innovation. The combination of these effects on supply (technology developers) and demand (potential users of technology) makes regulations a key issue in any country’s strategy for economic development.

The unnegotiable objective of establishing sanitary and phytosanitary regulations must be safety. Having said that, when different regulatory options provide an adequate level of safety, careful consideration should be given to select the option that is more likely to foster technological development, and thus avoid unnecessary brakes on the process of technological change (Ponte and Gibbon, 2005; Mancini, 2013; Tran et al., 2013).

According to a report by Moya-Angeler (2014), an increase in regulatory requirements usually hampers innovation by small enterprises, thus decreasing market competition, and ultimately driving a market concentration in large multinational companies (MNCs). This is particularly evident for regulations requiring extensive and expensive tests prior to the approval of a product, which discourage small and new innovative companies while granting a relative advantage to larger and established companies because they are better able to cope with the burden that this implies (Ashford and Heaton, 1983).

In this context, one of the current issues in development of agricultural biotechnology is analyzed next: the impact of regulatory requirements on innovations based on gene editing and other NBTs. An analysis of the Argentine experience may allow some conclusions to be drawn regarding the potential impacts on the agriculture and the biotechnology sectors. This would be a timely and valuable contribution to technology developers and policy makers in this area, as well as to the academic community working on “science and technology studies” (STS) (Hackett et al., 2008) particularly in the field of innovation economics.

## Comparative Analysis of GMOs vs. Gene-Edited Products Presented to the Regulatory System

### Timeline

**FIGURE 1:**
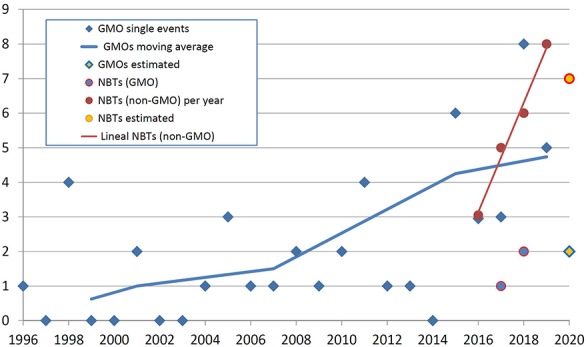
The timeline of GMO approvals in Argentina and the determination of conventional or GMO status for products obtained using different NBTs. The horizontal axis represents the year of the regulatory decision, and the vertical axis represents the number of products. See text for details.

Figure 1 exhibits the timeline of GMO approvals in Argentina *vis a vis* the determinations of conventional or GMO status for products obtained using different NBTs. It should be noted that the term “product” in this study is used for referring to cases where a regulatory determination has been made on an organism, and not necessarily refers to products that are actually available on the market.

It is important to note that the situation of a GMO being authorized is comparable with a determination that an NBT product is not a GMO. Both situations place the biotech crop at an equivalent instance, i.e., one step away from actual freedom to commercialize (that step being the registering of the product with the regulator of conventional products). Detailed comparison between the regulations for GMOs and conventional products including those obtained with NBTs in Argentina is provided in Whelan and Lema (2019).

In Figure 1, blue diamonds represent the number of new GMO single transformation events authorized per year since the first deregulation of an herbicide-tolerant soybean in 1996. The blue line is a moving average calculated on the basis of the period of Argentinean presidential terms of office (Wikipedia, 2020); this representation was included in order to help analyzing if there is a trend in the noisy data and, at the same time, to explore if there have been changes in public policy that might have influenced that trend. Finally, the yellow diamond for the year 2020 is an estimate based on the amount of GMO dossiers that have been filed recently and are currently under assessment.

Looking at NBTs in Figure 1, circles fully colored in red represent the number of NBT products that have obtained a determination of being non-GMO (i.e., conventional) organisms. The red line is a linear regression of such data; it was included to allow comparing with the changing slope of GMO approvals. The yellow circle for the year 2020 represents an estimate based on the number of informal inquiries that were attended recently.

Blue circles represent a few NBT products that were established to be GMOs; therefore, they should go through the GMO deregulation process, which would take several years for a subsequent approval. These cases were not considered further for the analyses presented next. For this reason, “NBT product” shall be understood as “non-GMO NBT product” for the remainder of this article.

Genetically modified organism approvals exhibit a trajectory that increases “noisily” but steadily. The noise at the yearly level is likely a consequence of assessing a time series made of small numbers that are the sum of few cases each year, and therefore it may be quite sensitive to particularities of individual cases. However, the moving average is always increasing, and it does not seem to be significantly affected by putative changes in biotech policies from one administration to the next. This average is likely growing in correlation with the generalized increase of traditional biotechnology development indicators, such as scientific publications, patents or R&D investment (Banerjee et al., 2000; Arundel, 2003; Reiss and Dominguez-Lacasa, 2016; OECD, 2019b).

In regards to NBTs, any insight from the very limited number of observations available shall be deemed preliminary. Having said that, it seems that NBT products, currently in the founding years, are emerging much faster compared with the foundational (or any other) period of GMOs. Roughly speaking, both product categories can be considered even now in terms of quantity of products arising per year, but if the apparent trends continue, NBTs will be significantly superior by numbers in the near future.

Although the same kind of comparison of relative development rates could have been made with the traditional indicators mentioned earlier, this measurement of “deregulation rate” is also enlightening, and perhaps even more useful to anticipate the actual use of these technologies in the field. This is because a comparison at the final stages of deregulation is obviously much closer to the actual market release compared with traditional indicators based on earlier stages of product development. Moreover, indicators based on advanced instances of deregulation are less likely to be skewed by proof-of-concept cases that ultimately were not destined to raise commercial interest.

### Developer Profiles

**FIGURE 2:**
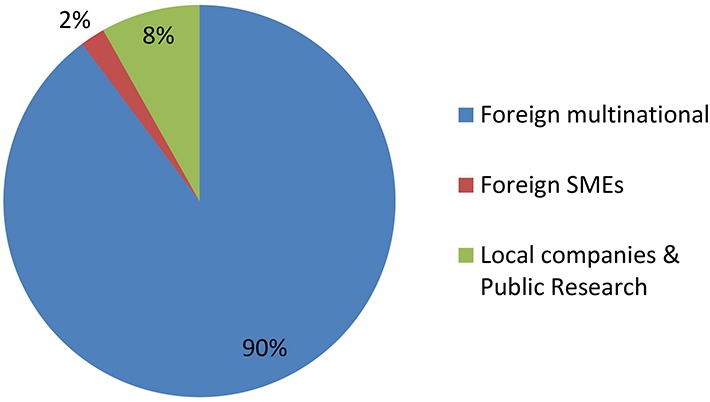
GMO products by developer profiles. See text for details.

**FIGURE 3:**
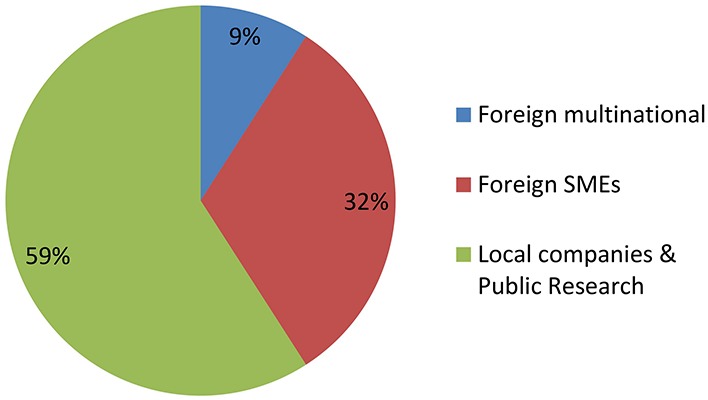
NBT (non-GMO) products by developer profiles. See text for details.

Figures 2, 3 shows groupings of the cases introduced in Figure 1 according to the developer’s profile. The criterion used to identify a MNC is taken from Dunning and Lundan (2008), while small and medium enterprises (SMEs) were classified as such according to internationally recognized criteria (OECD, 2019a,c). All foreign MNCs in this study have headquarters in developed countries. All Argentine companies in this study are SMEs with no subsidiaries, except for one multinational seed company with headquarters in Argentina (present in just 6 countries and quite small compared to the foreign MNCs).

Figure 2 shows that GMOs are deregulated mostly by MNCs, and actually such developers were the only group throughout the first two decades of the regulatory system. Only during the last 5 year has it been feasible that occasionally a local company or a foreign SME is able to deregulate a GM crop.

In contrast, Figure 3 shows that research institutes and/or local SMEs are responsible for about half of NBT products presented to the regulatory authorities, from the very beginning. In these cases, the whole process of product development, deregulation and commercialization is in the hands of such local actors from Argentina, a developing country. Regarding the other half of the cases, most of them correspond to products developed by foreign SMEs, and finally a small proportion was presented by MNCs.

### Number of Developers

**FIGURE 4:**
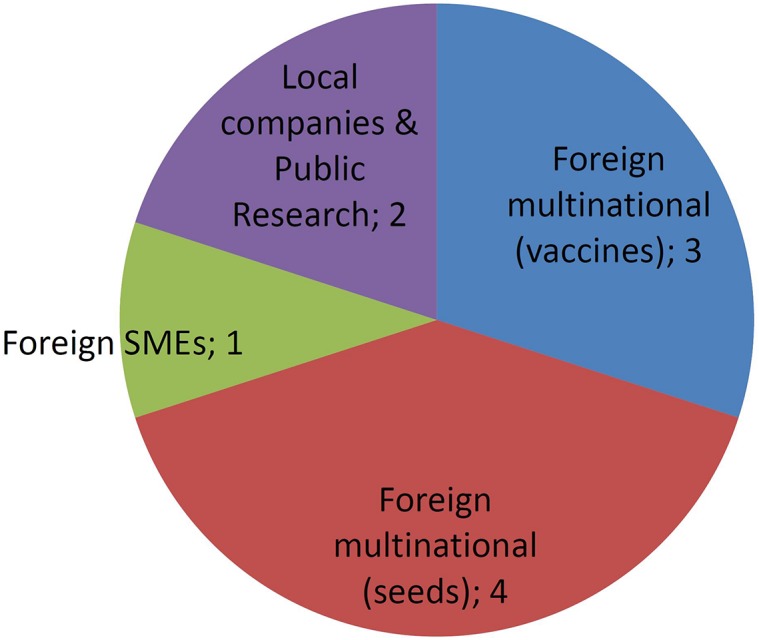
Numbers of each type of developers of GMOs approved. See text for details.

**FIGURE 5:**
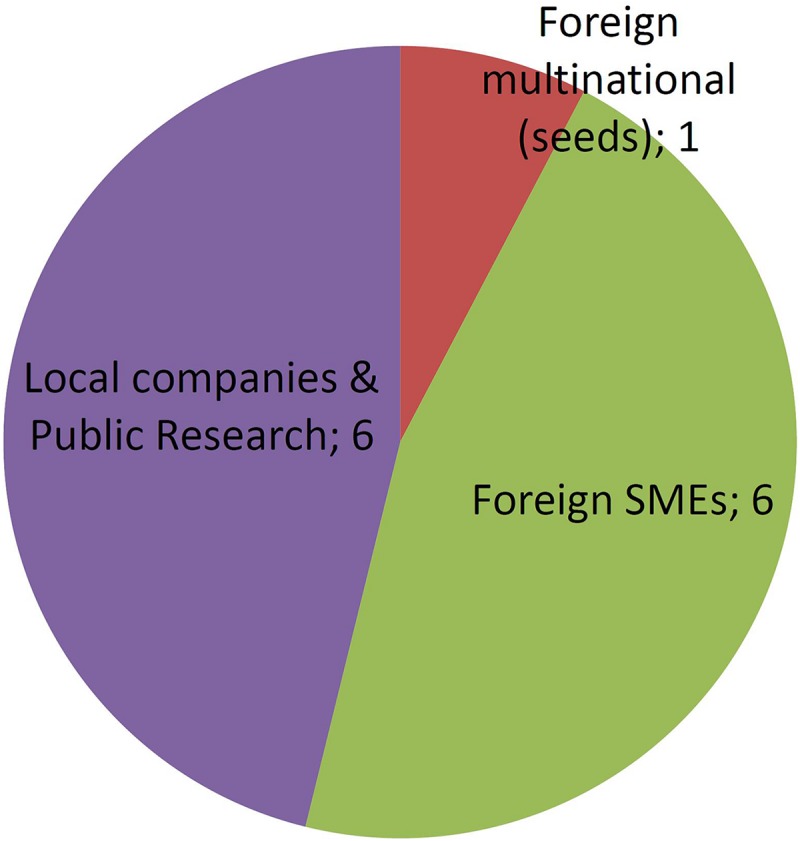
Numbers of each type of developers of NBT (non-GMO) products. See text for details.

Figures 4, 5 report the number of different developers (companies or institutions) corresponding to each one of the developer profiles as described previously. MNCs have been sub-divided into those commercializing veterinary vaccines or those dealing with GM crops. In regards to the latter, reckoning was based on currently existing business entities, thus taking into account the recurring processes of merging and acquisitions that took place during the last three decades in the field of GM crops.

Approved GMOs developed by MNCs are numerous, but concentrated in only four companies (Figure 4). In contrast, a few GMOs were deregulated by the public sector and SMEs and almost each one is owned by a different company.

Figure 5 shows that the number of different applicants for organisms improved using NBTs is already higher than the number of applicants that deregulated GMOs. This must be considered in perspective with the fact that NBT cases represent only a 3-year period of time, against a 23-year period for several dozens of GMOs.

In terms of product concentration, NBTs are typically distributed at 1–2 products per applicant, with only one outlier being an important Argentinean public research institute that holds 23% of applications. In contrast, the distribution of authorized GMOs per applicant is very uneven, with a handful of MNCs concentrating most products, including a single one that deregulated 40% of all GM crops.

From this insight, the market of crops and other agricultural organisms improved by NBTs is anticipated to be less concentrated in terms of proprietor entities. Therefore, it should be more competitive and more diversified, both in terms of commercialization conditions (cost, license conditions, etc.) as well as in regards to the availably of technical options in terms of traits and crops (the latter is explored next).

### Traits

**FIGURE 6:**
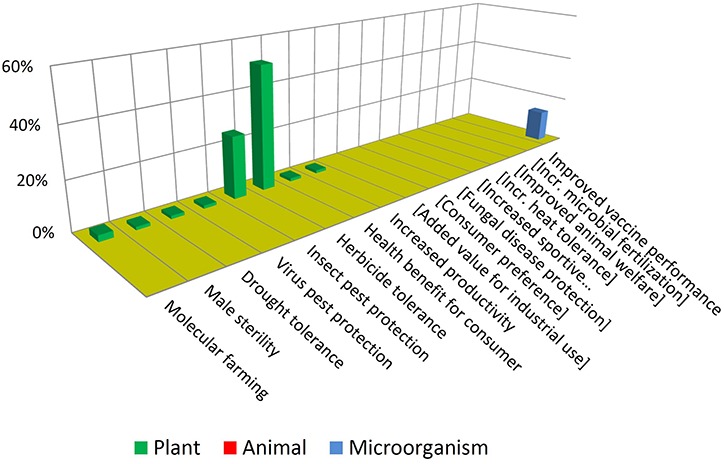
Introduced traits in GMO products. See text for details.

Figure 6 illustrates that most GMOs that have reached commercialization are plants having traits of herbicide tolerance and insect protection. Further to this, such traits are present mostly in three crops: maize, soybean and cotton. This situation is common to almost all countries growing GM crops (ISAAA, 2019).

Such products that consist in crops that are ubiquitously cultivated in large acreages combined with not-novel, unspecialized traits are sometimes referred to as “blockbusters” (Gewin, 2003; Stokstad, 2004). This expression captures the concept that MNCs tend to focus on conservative strategies involving crops and traits whose seeds may be demanded by farmers in high quantities and in many locations of the world. There are only a few “non-blockbusters” among approved GMOs. This includes drought tolerance, virus protection and even a case of “molecular farming” (Spiegel et al., 2018), consisting in a cheese-making enzyme produced in plants.

**FIGURE 7:**
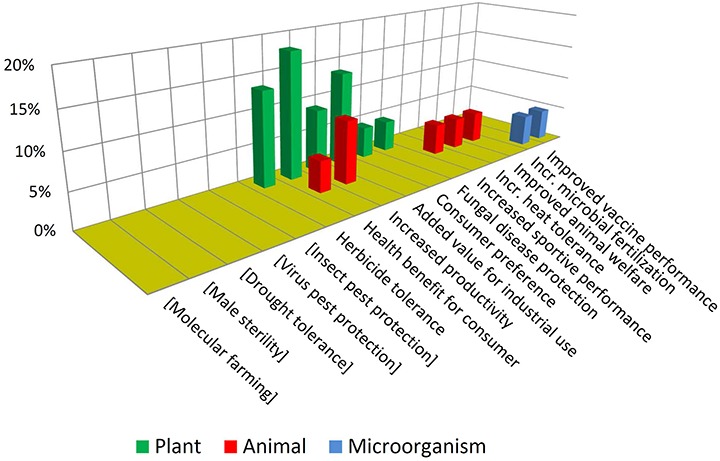
Introduced traits in NBT (non-GMO) products. See text for details.

In contrast with the above, Figure 7 shows that NBT products display a higher diversity in terms of traits and biological kingdoms. Such a difference may become bigger in the future, considering that the GMO cases are the result of a pipeline that has been stabilized over many years, while the unfolding of the NBT pipeline has begun much more recently.

Note that some traits which are not present among approved GMOs but are present among the NBTs have been included (enclosed in brackets) in Figure 6, and *vice-versa* in Figure 7, for a better comparison between the two figures.

It is also interesting to compare which traits are common or not to both groups. For instance, herbicide tolerance is significantly present for both technological options. This may be driven by its high demand as a blockbuster trait. In addition, for many crops there are no herbicide tolerant varieties, because of a lack of success regarding spontaneous mutations and “sociotechnical resistance” (Thomas et al., 2017) to GMOs; in such cases a gene-edited tolerant mutant may appear as promising alternative, worthwhile to be developed (Zhang et al., 2019).

In contrast, pest protection traits against insects and viruses, which are currently achieved by Bt proteins and RNA interference in GMOs, are not represented among NBT products; however, NBTs do include one case of protection against a fungus. This is a trait that has been repeatedly achieved by transgenesis but no GMO is commercially available yet; it has been suggested that the uncertainties and complexities of deregulating a fungus-protected GM crop have delayed such innovation (Cornelissen and Melchers, 1993; Wally and Punja, 2010). Perhaps in the case of NBTs a more affordable regulation would allow to reach the total investment required for delivering such kinds of traits to the market.

Drought tolerance is an intense field of development for both GMOs and NBTs (Cominelli and Tonelli, 2010; Jaganathan et al., 2018; Rodrigues et al., 2019), likely fostered by the increasing challenges derived from climate change. Although drought tolerance is currently represented only among GMOs, likely this will be also a target using NBTs, which nevertheless already includes one case pertaining to a different abiotic stress: heat tolerance.

Lastly in the case of molecular farming, such as industrial enzymes or pharmaceuticals produced in plants or animals, since this may only be possible by inserting genes from other organisms, such cases will always be considered GMOs.

### Distribution by Organism Type

**FIGURE 8:**
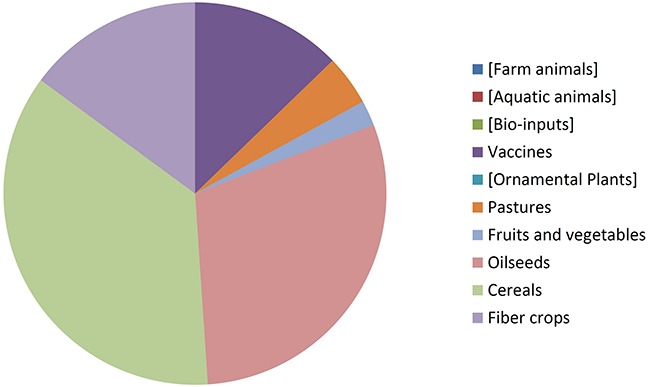
Approved GMOs distributed by type of organism. See text for details.

**FIGURE 9:**
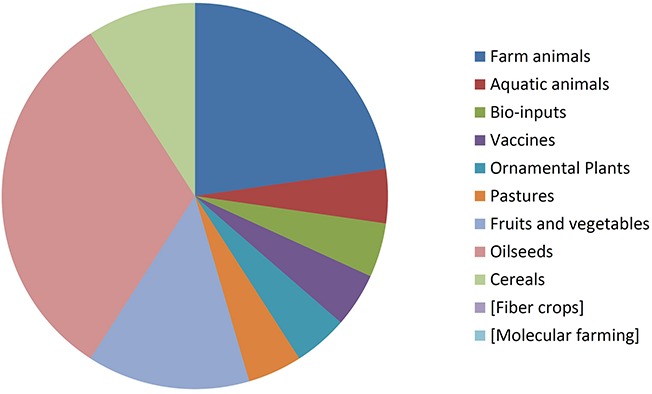
NBT (non-GMO) products distributed by type of organism. See text for details.

By comparing Figures 8, 9 it can be seen that diversity of organisms is already greater in NBT products than GMOs, grouped in terms of agricultural categories. This is because of differences in regards to (a) the presence of animals among the NBT cases, being absent among deregulated GMOs, (b) microbial products, where live and viable vaccines are present in both, but NBT products in addition include microbial agricultural “bioinputs” (Kour et al., 2017), and (c) diverse categories within the plant kingdom. Categories that are not represented in a figure but still shown for comparison with the other are enclosed in brackets.

Not surprisingly, GM crops are dominated by oilseeds, cereals and fiber crops, which in fact are represented by only one species each: soy, maize, and cotton. In contrast, albeit with lesser cases the NBT products are more dispersed among a higher number of crop categories and species. Interestingly, no fiber crops improved using NBTs have been presented yet. This might be expected, though, since cotton is a less-problematic kind of GMO in terms of trade issues and public perception, as it is a cash crop mainly used for obtaining non-edible textile material. Therefore, there might be less incentive for finding alternative innovative breeding technologies for cotton compared with other species.

### State of Development

**FIGURE 10:**
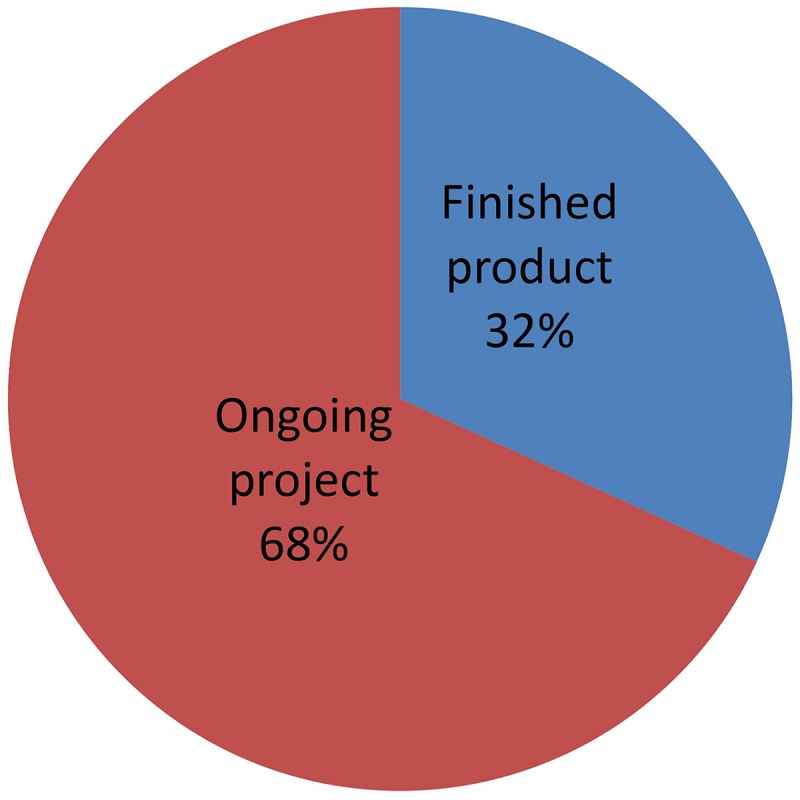
Distribution of NBT (non-GMO) products, classified by state of development. See text for details.

Figure 10 shows a distribution of NBT products that have been submitted to the Argentine regulatory system, classified according to their level of development. “Finished product” means those whose breeding process is complete and the product has been fully studied at the phenotypic and molecular levels. Such products are in a position to receive a final determination of “non-GMO” status.

Conversely, “Ongoing projects” are those where the final characterization of the product is not fully available. As described by Whelan and Lema (2015), developers at this stage are able to request a formal preliminary analysis based on the expected characteristics of the final product, which shall be re-confirmed later when a full phenotypical and molecular characterization becomes available.

Many developers are requesting this option of preliminary analysis. This is presumably because they find it very valuable for planning and taking decisions on continuing with the project, as well as for attracting funding once they can estimate the regulatory costs with more reliability. The option of receiving a formal preliminary analysis is likely playing an important role in fostering investment and development of NBT products.

### Usage of Gene-Editing Within NBTs

**FIGURE 11:**
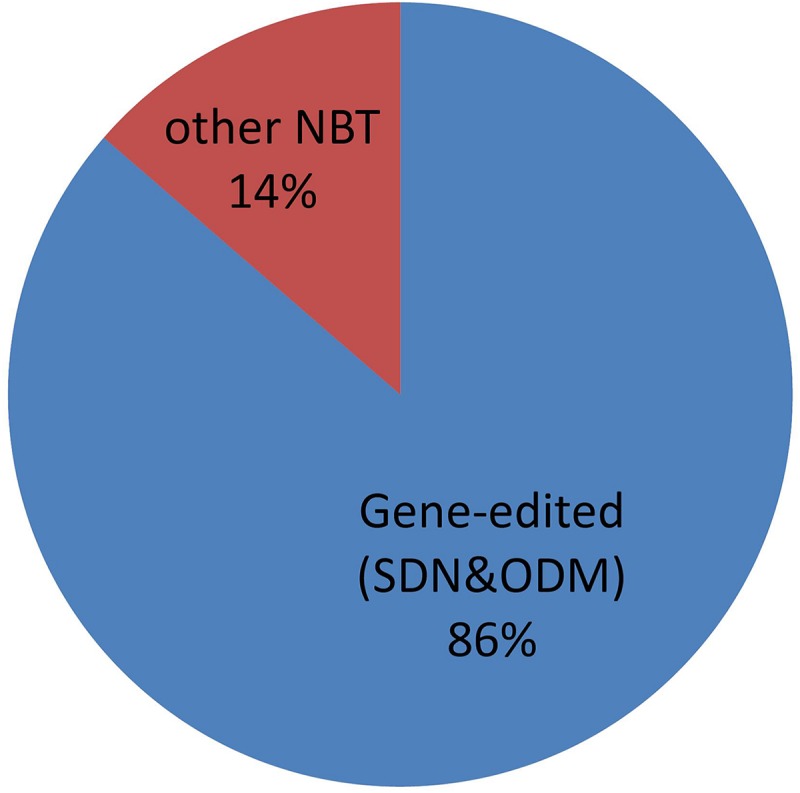
Usage of Gene-editing within NBTs. See text for details.

Gene editing, especially using CRISPR-Cas nucleases, is attracting a lot of interest for breeding and other purposes (Jaganathan et al., 2018; Chen et al., 2019). Argentine regulation for NBTs of course includes products obtained by genome editing and, not surprisingly, it is the most commonly applied NBT of the cases submitted to the regulatory system in Argentina. See Figure 11.

For this analysis, we have considered gene editing to include techniques encompassed by the terms “site-directed-nuclease” (SDN) of types 1, 2, and 3, as well as “oligonucleotide-directed-mutagenesis” (ODM), according to the definitions by Lusser et al. (2011). Counter-examples of NBTs that are not gene editing techniques include epigenetic modification (Álvarez-Venegas and De-la-Peña, 2016), reverse breeding (Dirks et al., 2009), etc.

Although genome editing represents a vast majority, there is also a proportion of other NBT products. It is important to realize that this is a rapidly evolving field, where regulation must be designed to withstand the test of time (i.e., technical advances) as much as possible. As a demonstration of this, it can be pointed out that the term “NBT” was coined -for regulatory purposes- 1 year *before* the first CRISPR-Cas tool became known, but nowadays it has become the dominant technology within NBTs. Novel gene editing techniques are published and patented every month, and their similarities and/or differences with other NBTs are more difficult to define, for instance with CRISPR-Cas tools adapted to perform epigenetic interventions (Pickar-Oliver and Gersbach, 2019).

In this sense, it is important to highlight that the Argentine regulation has been scripted without the need of inserting a list of specific techniques. Consequently, it is not restricted to the particular technological configurations available at the time the regulation was drafted. Therefore, it avoids delaying or discouraging incremental innovations as they appear later on.

## Conclusion

This article has compared apparent trends amongst technologies presented to the Argentine regulatory system for agricultural biotechnology. This was done with the purpose of detecting emerging opportunities for strengthening local innovation processes in the agricultural sector. This is just an initial study, because further STS are needed for a more broad and comprehensive research agenda on innovations enabled by gene editing and other NBTs. Such an agenda should include (a) comparative case studies of specific products having the same trait but obtained through different breeding technologies (such as Bullock et al., 2019), as well as (b) quantitative estimations of the macroeconomic impacts derived from NBT products altogether.

According to the preliminary evidence presented here, the regulatory approach adopted in Argentina is already stimulating local innovation processes. Noticeable changes include an increase of technology developers/providers and the diversification of products; the potential impacts appear to be higher for breeding niches that have not been explored yet by (commercial) agricultural biotechnology.

It has been postulated already that genome editing will be a democratizing technique; however, these assertions were based on qualitative reasoning or very early milestones of technology development (Jackson et al., 2019). In this work we present evidence for this trend that is collected closer to the actual use of this technology. A corollary is that genome editing should be less prone to the criticism/protectionism raised against GM crops from allegations that they could affect “food democracy” (Friedrich et al., 2019) or food security/sovereignty.

Moreover, it can be proposed that a reasonable regulation for gene editing, in particular, will have an immediate and direct effect on the agricultural innovation system, particularly if it allows improving the predictability of regulatory costs for innovative products. Besides this, the investment of time and money required in order to meet regulatory requirements may be more attainable compared with the option of developing the same traits using GMO technology.

Gene editing is perhaps the newest paradigm shift of the present-day industrial revolution that encompasses biotechnology (Rifkin, 1998; Karan, 2016). The emergence of a technological paradigm creates a context for establishing new development policies that expand opportunities for local actors (Freeman and Pérez, 2003). Taking into account that opportunities for economic development are a mobile target, sometimes linked to paradigm shifts (Pérez, 2004), and genome-edited products constitute a window of opportunity for developing countries. This opportunity is also available to developed countries where the first wave of local development based on GMOs crashed against a barrier of over-regulation (Jorasch, 2019). Not surprisingly, the forerunner Argentine regulation has inspired another eight countries in Latin America to enact similar regulations in less than 4 year, and is quite in line with regulatory developments occurring recently in countries from Africa, Asia and Oceania.

A more dynamic market of innovation creates opportunities to expand the supply of local technologies. This can strengthen the agricultural innovation system, because it allows new actors to enter through the window of opportunity. The technological shift makes it easier for SMEs and public R&D laboratories to develop new products on their own, thus expanding the market, both in terms of participants and products. In addition, the reduction in the scale of production necessary to reach profits can favor the development of local economies.

In conclusion, the results of this prospective study suggests that gene editing could drive further innovation and “democratization” of agricultural biotechnology, thus leading to increased productivity and economic development, if managed under effective regulatory processes.

## Data Availability Statement

The datasets generated for this study are available on request to the corresponding author.

## Disclaimer

The information and views are those of the authors as individuals and experts in the field, and do not necessarily represent those of the organizations where they work.

## Author Contributions

All authors listed have made a substantial, direct and intellectual contribution to the work, and approved it for publication.

## Conflict of Interest

The authors declare that the research was conducted in the absence of any commercial or financial relationships that could be construed as a potential conflict of interest.
